# Wound healing in the jugal mucosa of rats with a cold blade scalpel and an ultrasonic harmonic scalpel

**DOI:** 10.1016/S1808-8694(15)30652-2

**Published:** 2015-10-19

**Authors:** Elen Carolina David João De Masi, Sergio Luis Rocha, Marcos Mocellin, João Luis Garcia de Faria

**Affiliations:** 1 MSc in Surgery. Otorhinolaryngologist; 2 PhD in Surgery. Adjunct Professor - Federal University of Paraná (UFPr) and Full Professor of the Catholic University of Paraná (PUCPr); 3 PhD in otorhinolaryngology, Head of the Otorhinolaryngology department and full processor - UFPr; 4 PhD in otorhinolaryngology, Adjunct Professor of the Otorhinolaryngology department - UFPr

**Keywords:** wound healing, surgery, oral mucosa

## Abstract

Ultrasound harmonic scalpel has been recently introduced in otorhinolaryngological procedures.

**Aim:**

to assess macro and microscopic evolution of the healing process of wounds created in the jugal mucosa of rats by the use of ultrasound scalpel.

**Method:**

we used 30 Wistar rats in which we made mucosal incisions on the right jugal mucosa with the ultrasound harmonic scalpel (USHS) and on the left side with the cold blade scalpel (CBS). Macroscopic and microscopic evaluations were carried out on the third, seventh and fourteenth days of postoperative. For the microscopic evaluation we used HE to asses the inflammatory process and the Sirius Red approach for collagens type I and III. Anti-CD 3 antibodies and anti-factor VIII assessed the concentration of T-lymphocytes and neovessels.

**Results:**

the USHS caused greater cell damage with reepitelization delay. Microscopy showed more intense inflammatory reactions and a loss in collagen build up, delay in scar maturation and a greater vessel neoformation.

**Conclusion:**

USHS brings about a greater lesion in the incision area; delayed regeneration; promotes greater inflammatory process and angiogenic activity; delays in fibroplasia and scar tissue maturation on the rats' jugal mucosa when compared to cold blade scalpel.

## INTRODUCTION

The basic principles of surgery involve three pillars: dieresis, synthesis and hemostasis^1^. Many heat-producing devices were developed and introduced in surgical practice in recent years in order to improve hemostasis, with minimum tissue damage during operations. These devices include electrocauteries, radiofrequency and CO_2_ laser, which use thermal energy to denaturate protein, causing vascular packing and consequent hemostasis; however, they have drawbacks because of lateral heating in the operating field, which can damage structures adjacent to where it is deployed and thus delay wound healing^2^.

Today, among the differentiated methods available regarding incision and hemostasis, we stress the harmonic ultrasound scalpel (HUS), radiofrequency, electrocautery and the laser beam^3^.

Tissue heating up caused by HUS has minimum depth of penetration when compared to laser, radiofrequency and electrocautery, with reduced tissue damage^4^. Metternich et al.^5^,^6^ showed the macroscopic difference in mucosal healing in wounds made by cold blade scalpel (CBS) and HUS; nonetheless, these papers point towards the ease of using these tools and very few of them report the results of the effects in the healing process. Thus, experimental histopathology studies become important in the follow up of scar formation process in oral cavity lesions treated with this type of tool. In order to assess the macroscopic and microscopic evolution of the healing process of wounds created with the HUS on the jugal mucosa, we developed this experimental study in rats.

## MATERIALS AND METHODS

### Study Design

Retrospective study of the healing process involving 30 Wistar rats (Rattus norvegicus albinus, Rodentia mammalia). The study was approved by the Ethics in Research with Animals Committee from the Catholic University of Paraná, under protocol # 69, in compliance with federal law # 6638 and the guidelines from the Brazilian College of Animal Experimentation (COBEA). We used 10 rats in each assessment period (third, seventh and fourteenth days), chosen randomly.

### Procedure

The surgical procedure was carried out with the animals under general anesthesia by intramuscular injections of ketamine and xylazine. All the animals suffered a longitudinal incision on their jugal mucosa of both sides of their mouths, using the CBS on the left side and the HUS on the right side. As point of reference we used the labial slot, with the incision being extended in one centimeter in length and depth until the muscle layer. The wounds were not sutured and healing happened by second intention. We used oral acetaminophen in a single 200 mg/kg dose in the postoperative.

Clinical evaluation was carried out in the immediate post-op and on the third, seventh and fourteenth days of post-op. Immediately after the incision, we noted the presence or absence of bleeding and when present, it was classified as mild when there were signs of blood on the wound, moderate when it required compression to stop the bleeding, and intense when, even under compression, bleeding remained. As far as the macroscopic evolutional evaluation of the wounds is concerned, we noted whether they were open or closed, broad or narrow and with or without granulation tissue. On the closed wounds, we noted whether they were depressed or invisible. The animals were randomly chosen and assigned to groups of 10 each, were slaughtered according to the scheduled time of post-op through an intraperitoneal injection of sodium phenobarbital, at a dose of 120 mg/kg (resolution 714 from the Federal Board of Veterinary Medicine).

### Histopathological Analysis

After the slaughter, the jugal mucosa scar from both sides were removed in a spindle-like fragment and fixed in 10% formaldehyde for 24 hours. After processed, they were dyed by hematoxylin-eosin (HE). The morphological analysis was done at three given times, in other words, third, seventh and fourteenth day after the incision.

We assessed the number of predominant cells in the inflammatory reaction, the presence of interstitial edema, vascular congestion, level of granulation tissue formation and fibroplasty, microabcess and necrosis.

The morphometric analysis was carried out only on the seventh and fourteenth days, since the markers were geared towards the chronic phase of inflammation and healing.

Sirius red was used to assess collagen. Immunohistochemistry was carried out with anti-factor VIII and anti-CD3 antibodies in order to count the neoformed vessels and T-lymphocytes, respectively.

The statistical analysis used to compare the two types of scalpel was carried out by the Wilcoxon non-parametric test, binomial test and the Mann-Whitney non-parametric test.

## RESULTS

**Figure 1:**
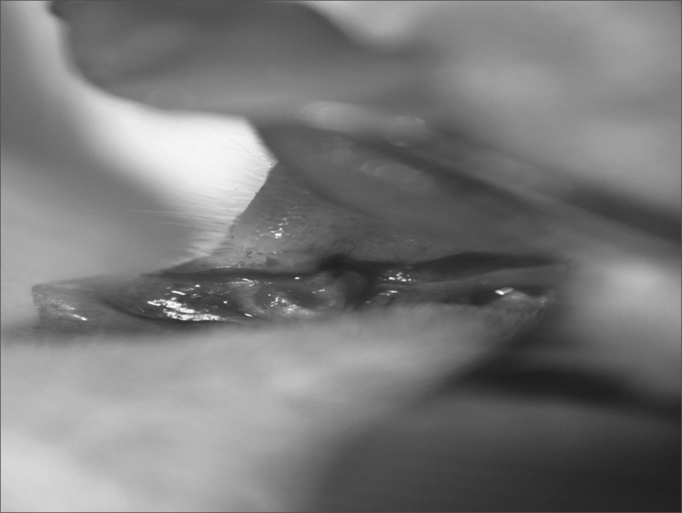
Immediate aspect of the CBS incision.

**Figure 2:**
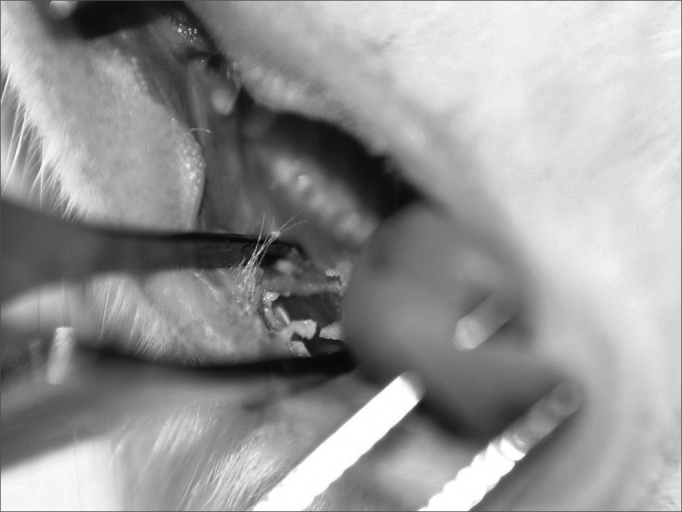
Immediate aspect of the HUS incision.

One animal died in the immediate post-op because of intense bleeding from the incision made by the CBS. Still in immediate observation of the 30 rats, incisions made with the CBS was narrow and the ones made with the HUS were broad (Figure 1, Figure 2). On the third day, the wounds made with the HUS were larger and more depressed, and they all had granulation tissue. On the seventh day, all the CBS incisions were closed and leveled off, while those made with the HUS were open, broad, depressed and with granulation tissue. On the fourteenth day, the scars from the incisions made with the CBS were invisible, and those made by the HUS were not totally epithelized.

**Figure 3:**
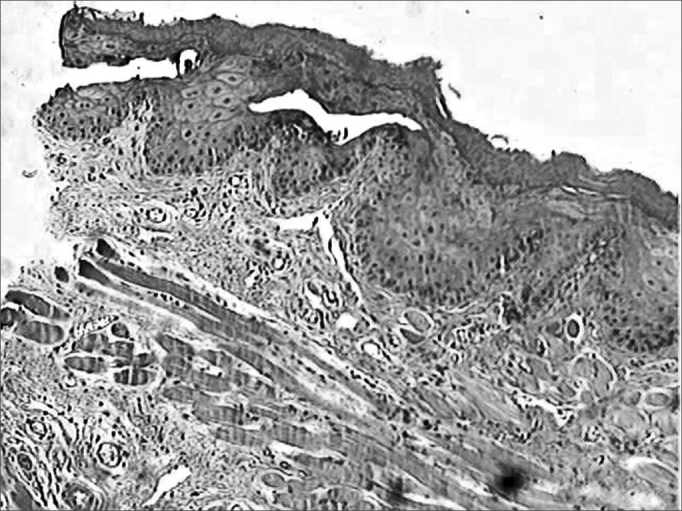
Microphotographies of histological cross-sections showing the presence of inflammatory process on the third day of evolution, on the wounds made with the CBS (HE, 100 X).

**Figure 4:**
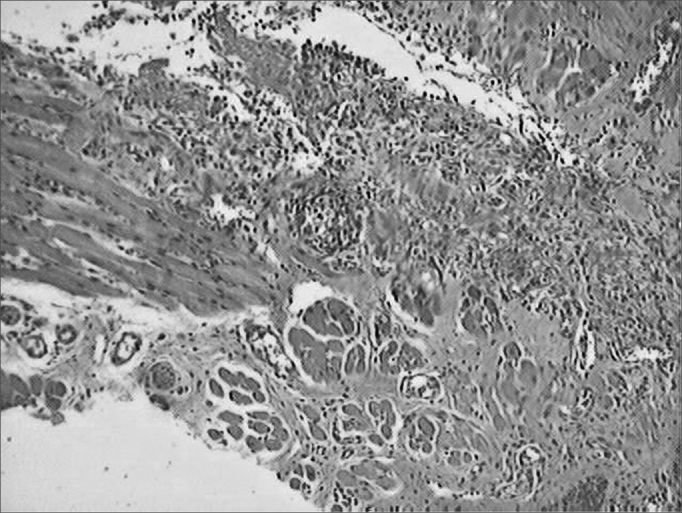
Microphotographies of histological cross-sections showing the presence of inflammatory process on the third day of evolution, on the wounds made with the HUS (HE, 100 X).

**Figure 5:**
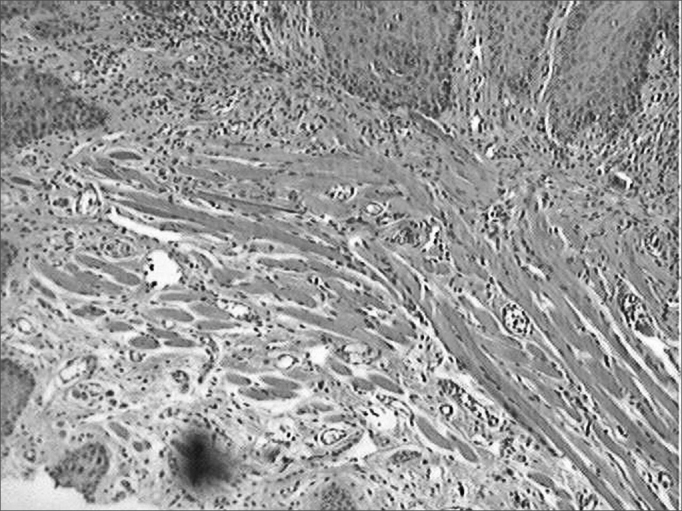
Microphotographies of histological cross-sections showing the presence of inflammatory process on the seventh day of evolution, on the wounds made with the CBS (HE, 100 X).

**Figure 6:**
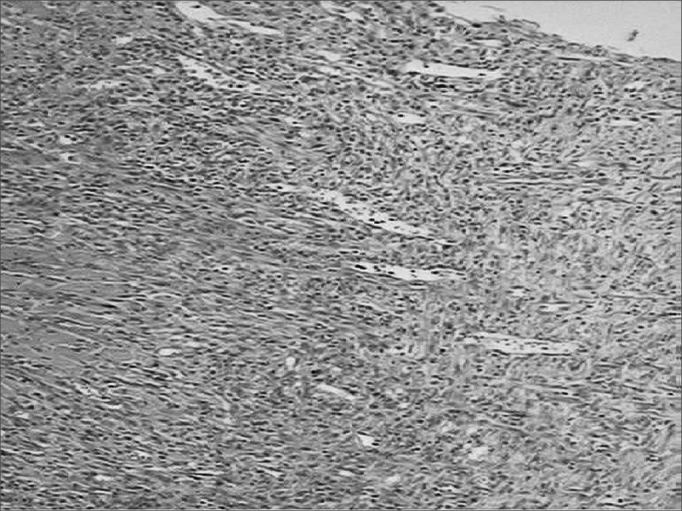
Microphotographies of histological cross-sections showing the presence of inflammatory process on the seventh day of evolution, on the wounds made with the HUS (HE, 100 X).

**Figure 7:**
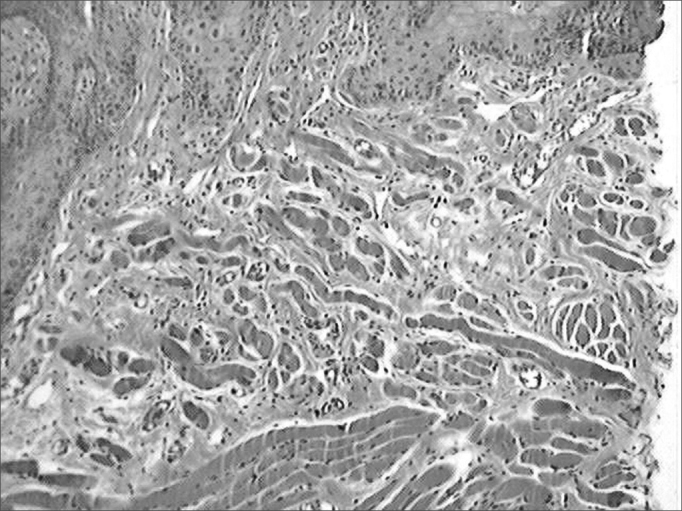
Microphotographies of histological cross-sections showing the presence of inflammatory process on the fourteenth day of evolution, on the wounds made with the CBS (HE, 100 X).

**Figure 8:**
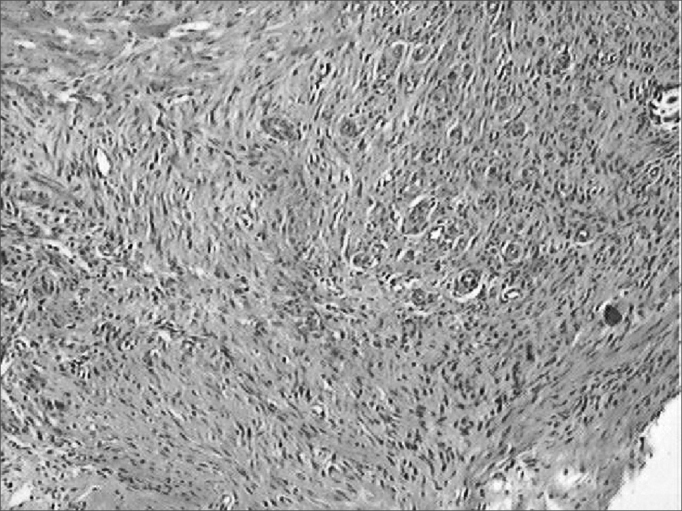
Microphotographies of histological cross-sections showing the presence of inflammatory process on the fourteenth day of evolution, on the wounds made with the HUS (HE, 100 X).

During the microscopic analysis carried out on the third day, both wounds had acute inflammatory processes. On the seventh day, on the incisions made with the CBS, wound healing was complete and with chronic inflammatory process. On the incisions made by the HUS, the wounds were still under acute inflammatory process. On the fourteenth day, the histological cross sections of the scars of incisions made by the CBS showed complete regeneration, while those incisions made with the HUS, showed a chronic inflammatory process (Figure 3, Figure 4, Figure 5, Figure 6, Figure 7, Figure 8).

Considering morphometric microscopy, collagen evaluation happened on the seventh and fourteenth days. Collagen intensities on the seventh day were greater in the wounds created by the CBS for both fractions I (p=0.005) and III (p=0.037) (Table 1). On the fourteenth day, the values maintained favorable to CBS for collagen I (p=0.008); however, with greater intensity for collagen III with HUS (p=0.038) (Table 2).

**Table 1:** Comparing the collagen I and III variables on the seventh day.

Variable	Scalpel	N	Mean	Median	Minimum	Maximum	Standard deviation	*p value
Col. I	CBS	10	4,65	3,07	1,63	10,17	3,22	0,005
	HUS	10	0,89	0,79	0,65	1,39	0,28	
Col. III	CBS	10	2,85	2,06	1,01	6,11	1,84	0,037
	HUS	10	1,44	1,28	0,8	2,49	0,54	

* Wilcoxon non-parametric test

**Table 2:** Comparing collagen I and III variables on the 14th day.

Variable	Scalpel	N	Mean	Median	Minimum	Maximum	Standard deviation	*p value
Col. I	CBS	9	2,71	2,59	0,83	5,18	1,69	0,008
	HUS	10	0,85	0,67	0,39	2,15	0,55	
Col. III	CBS	9	1,3	0,68	0,33	3,26	1,04	0,038
	HUS	10	2,52	2,6	1,51	3,9	0,9	

* Wilcoxon non-parametric test

The analysis with anti-CD3 on the seventh day revealed a greater number of T-cells on the wounds made by the HUS (p= 0.005), on the fourteenth day, the concentration of these cells was similar in both wounds (Table 3). The analyses with anti-factor VIII on the seventh and on the fourteenth days of post-op revealed a greater number of neovessels on the wounds created by the HUS, with a p=0.005 for the seventh and fourteenth days (Table 4).

**Table 3:** Comparing T-lymphocyte concentrations detected by anti-CD3 on the 7th and 14th days.

Variable	Scalpel	N	Mean	Median	Minimum	Maximum	Standard deviation	*p value
CD3	CBS	10	29	28,5	19	41	7,93	0,005
7th d	HUS	10	107,4	104,5	79	147	21,98	
CD3	CBS	10	19,5	19,5	14	27	4,35	0,327
14 th d	HUS	10	25,5	21	11	57	16,09	

* Non-parametric Wilcoxon test

**Table 4:** Comparing the number of vessels assessed by the anti-factor VIII on the 7th and 14th° days.

Variable	Scalpel	N	Mean	Median	Minimum	Maximum	Standard deviation	*p value
F VIII	BLF	10	2,4	2,5	1	4	1,17	0,005
7th d	BHU	10	12,1	12,5	9	15	2,13	
F VIII	BLF	10	0,3	0	0	1	0,48	0,005
14th d	BHU	10	5,3	5	4	7	1,16	

* Non-parametric Wilcoxon test

## DISCUSSION

HUS is a tool that cuts and causes hemostasis at the same time. It has been broadly used in video-assisted surgeries and recently is has been used in otolaryngological and head and neck surgeries. Its properties are advocated by numerous authors^3^, ^4^, ^5^, ^6^, ^7^, ^8^, ^9^, ^10^, ^11^, ^12^, ^13^. In the study we hereby present, there was a trend towards a better hemostasis caused by the HUS, because the incisions made by such device did not bleed. Nonetheless, on the wounds caused by the CBS, one animal died because of bleeding. The wounds created by the CBS were narrower and on the seventh day were already epithelized, and this did not happen completely until the fourteenth day on HUS wounds^14^. It is very likely that the delay in the process could be explained by the fact that, even designing lesions of the same size, after their completion, the incisions made by the HUS resulted in broader wounds when compared to those created by the CBS. Sinha and Gallagher^2^ showed that in the oral mucosa of guinea pigs, the HUS caused a longer and more intense inflammatory process and confirmed this information in all the stages of the analysis. HUS showed a greater inflammatory process in relation to the CBS, and such fact is understandable, since the incisions made with the CBS resulted in regular and well outlined margins, while those resulting from the HUS were wider and with more tissue damage, which caused a longer and more intense inflammatory process during healing. Considering that the inflammatory reaction was longer and more intense on the wounds created with the HUS, it was to be expected that there would be a delay in relation to fibroplasty^3^,^14^,^15^. In the present investigation we noticed that the collagen density found in the histological cross-sections was greater in the wounds resulting from the CBS on the seventh day of evaluation. Moreover, on the tenth day we found a greater amount of type III collagen on the wounds resulting from the HUS incision, showing a delay in scar maturation process. Immunohistochemical analysis showed a greater and longer inflammatory process associated on the wounds made by the HUS^15^ because of the number of T-cells and neovessels found. Although the incisions made by HUS cause a delay in the healing process when compared to those produced by CBS, and confirmed in this study by the clinical, microscopic, morphological and morphometric evaluations, the HUS is a cutting and hemostasis-causing tool at the same time, with low production of thermal energy on the tissues, providing a cleaner and faster surgical procedure thanks to the lack of bleeding. Further studies must be carried out in order to prove the effects of temperature on the tissues in relation to depth, because other types of electrical scalpels and laser transmit too much heat, contrary to the HUS which produces less heat when compared to these other devices.

## CONCLUSION

Incisions with HUS made on the jugal mucosa of rats caused a delay on the regeneration process, promoted a more prolonged and intense inflammatory process, developed greater angiogenic activity and delays in fibroplasia and scan maturation, when compared to incisions made with the CBS.
